# Printed Large-Area Single-Mode Photonic Crystal Bandedge Surface-Emitting Lasers on Silicon

**DOI:** 10.1038/srep18860

**Published:** 2016-01-04

**Authors:** Deyin Zhao, Shihchia Liu, Hongjun Yang, Zhenqiang Ma, Carl Reuterskiöld-Hedlund, Mattias Hammar, Weidong Zhou

**Affiliations:** 1 Department of Electrical Engineering, University of Texas at Arlington, TX 76019, USA; 2 Department of Electrical and Computer Engineering, University of Wisconsin-Madison, WI 53706, USA; 3 KTH-Royal Institute of Technology, School of Information and Communication Technology, Electrum 229, 164 40 Kista, Sweden

## Abstract

We report here an optically pumped hybrid III-V/Si photoic crystal surface emitting laser (PCSEL), consisting of a heterogeneously integrated III-V InGaAsP quantum well heterostructure gain medium, printed on a patterned defect-free Si photonic crystal (PC) bandedge cavity. Single mode lasing was achieved for a large area laser, with a side-mode suppression ratio of 28 dB, for lasing operation temperature ~200 K. Two types of lasers were demonstrated operating at different temperatures. Detailed modal analysis reveals the lasing mode matches with the estimated lasing gain threshold conditions. Our demonstration promises a hybrid laser sources on Si towards three-dimensional (3D) integrated Si photonics for on-chip wavelength-division multiplex (3D WDM) systems for a wide range of volume photonic/electronic applications in computing, communication, sensing, imaging, etc.

Si photonics using CMOS-compatible processes have made great advances through the development of Si-based photonic devices^1^,^2^,^3^. One of the most active and challenging research areas remains to be related to the on-chip lasers, with the most promising approach being heterogeneous integration of direct-bandgap group III-V materials on Si^1^,^4^,^5^,^6^,^7^,^8^,^9^,^10^,^11^ through wafer bonding, printing, and direct-growth. Most lasers demonstrated so far are edge-emitting lasers, the dominant light sources used in high speed, high capacity WDM optical communication systems, owning to its excellent high power operation and stable single mode spectral properties especially in DFB laser cavities. On the other hand, despite their advantageous properties, such as circular beam shape, catastrophic optical damage free, and two-dimensional integration capabilities with CMOS electronics, vertical-cavity surface-emitting lasers (VCSELs) are largely limited to applications in data-com, computing, and consumer electronic systems, owing to its relatively low output power (limited by the small cavity volume) and relatively poor spectral (linewidth and RIN) properties. The realization of high power, energy efficient single-mode operation with a single-lobe beam in VCSELs faces difficult challenges related to transverse mode control with relatively large aperture sizes. Photonic crystals (PC) have been incorporated in the laser cavity design in addressing these issues, along with the demonstrations of photonic crystal defect-mode lasers and photonic crystal confined VCSELs^12^,^13^,^14^,^15^,^16^,^17^,^18^,^19^,^20^,^21^.

The bandedge effect in defect-free photonic crystal structures, where the group velocity is close to zero, has advantages in achieving high performance semiconductor surface emitting lasers, including low lasing threshold, single longitudinal and transverse mode over a large lasing area, narrow linewidth, high power output, small beam divergence angle, polarization control, and output beam pattern control^18^,^21^,^22^,^23^,^24^,^25^. Based on the bandedge effect, high performance lasers on GaAs has been demonstrated, including a very recent demonstration of high power, single mode, large area PC bandedge lasers with unpatterned QW active region using a regrowth process^21^.

Based on the membrane transfer printing technique for heterogeneous integration of III-V with Si materials^26^, we demonstrate printed large-area single-mode photonic bandedge lasers on Si. Different from the membrane lasers reported earlier^26^, the structure reported here does not require a low index buffer layer to be placed in between the active region and the Si PC layer. It only requires a one-step printing process for the heterogeneous integration of III-V gain structure on top of the patterned Si PC cavity. The printing process also enables the possibility of different gain structures to be printed on the top of the same Si chip for wide spectral coverage and multi-band WDM on-chip. Single mode lasing operation was demonstrated from large area cavities at 1550 nm spectral band, with very narrow linewidth and high side-mode suppression ratio (SMSR). It is worth noting that currently the lasing performance is largely limited by the transfer printing process. With further optimization of the transfer printing process, and precise control of the interface quality, higher performance lasers are feasible.

## Cavity design and fabrication

**Figure 1:**
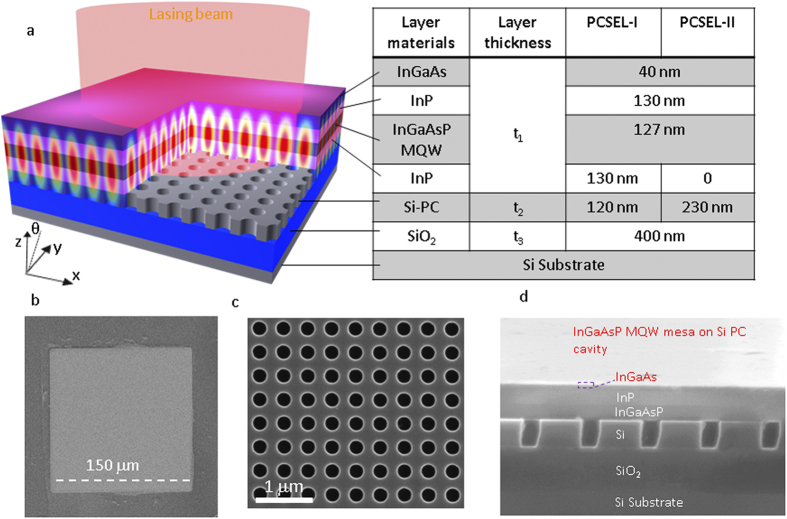
PCSEL on silicon. (**a**) Schematic of hybrid III-V/Si PCSEL cavity on Si substrate, which consists of III-V active MQW structure and Si-PC cavity. Also shown is the simulated electrical field distribution in the cavity for a lasing mode at 1424 nm. (**b**) A SEM image of InGaAsP QW disk/mesa transferred onto a Si-PC. (**c**) Zoomed-in view of defect-free Si-PC cavity. (**d**) Cross sectional view of the cavity.

As shown schematically in Fig. 1(a), the lasing cavity consists of a transferred III-V InGaAsP multi-quantum well (MQW) heterostructure active region, printed on a single-layer Si-PC cavity. Also shown in Fig. 1(a) is the simulated optical magnetic H-field distribution profile for the designed lasing cavity mode. In such cavities, patterned PC structure was designed to be placed very close to the unpatterned QW structure to provide strong in-plane distributed feedback. The lightwaves propagating in various 2D directions are coupled with one another, and a 2D standing wave or 2D cavity mode is constructed over a broad area^21^. The in-plane propagating waves are also diffracted toward the vertical direction by the PC structure itself due to the first-order Bragg diffraction^19^.

Si-PC cavity was first designed based on a silicon-on-insulator (SOI) substrate, with a top Si layer thickness (t_2_) of 230 nm and a buffered oxide (BOX) layer thickness (t_3_) of 400 nm. Total thicknesses of the top InGaAsP MQW heterostructure (t_1_) and the bottom Si-PC cavity (t_2_) vary for two device designs operating at different temperatures, as shown in the structure table in the inset of Fig. 1(a), denoted by PCSEL-I and PCSEL-II, respectively. The lattice constant for the square lattice air hole Si-PC lattice is chosen to be *a* = 480 nm. To match InGaAsP MQW emission peak locations at different operation temperatures, the air hole radii are *r* = 0.2*a* and *r* = 0.15*a* for PCSEL-I and PCSEL-II, respectively.

The whole InGaAsP MQW structure was grown on (001) n-InP substrate by metal-organic chemical vapor deposition (MOCVD), consisting of top and bottom InGaAs contact layer, top and bottom InP cladding layers, and eight pairs of strain-compensated In_0.76_Ga_0.24_As_0.83_P_0.17_/In_0.485_Ga_0.515_As_0.83_P_0.17_ with the center emission wavelength of 1,527 nm at room temperature^27^. A InGaAs/InP sacrificial bilayer was grown first on InP substrate for selective wet-etching and substrate removal. Total thickness (t_1_) is 427 nm for device PCSEL-I and 297 nm for device PCSEL-II. For the PCSEL-II device, the bottom InP cladding layer were selectively etched away for stronger evanescent coupling between the MQW and the Si PC cavity. Accordingly, for PCSEL-I, the Si-PC layer was first thinned down from 230 nm to 120 nm based on thermal oxidation and selective wet etching processes.

The 2D 0.5 × 0.5 mm^2^ square lattice PC pattern was fabricated based on the standard e-beam lithography (EBL) patterning technique and plasma dry-etching processes. Substrate removal process is used here for the release of top large area InGaAsP MQW structure from the native InP substrate with black wax as the protection layer on top. InP substrate was removed by immersing the sample in 20%HCl + 80%H_3_PO_4_ for about two hours. The active region was then lifted off as a membrane by selective wet etching to release the bottom InGaAs/InP sacrificial bi-layers and the InGaAs n-contact layer. For PCSEL-II, the bottom InP n-cladding layer was also selectively etched away. The released InGaAsP MQW membrane is finally printed onto the patterned Si PC cavity. Wax was removed with trichloroethylene (TCE) and IPA solutions. At the end, a 150 × 150 μm^2^ active MQW square area is defined by optical lithography alignment and etching process, with alignment accuracy around 1–2 μm. Figure 1(b) shows the SEM top view of a printed InGaAsP QW membrane on a Si PC cavity on a SOI substrate, with a zoomed-in view and a cross-sectional view shown in Fig. 1(c,d), respectively.

## Device characterizations

**Figure 2:**
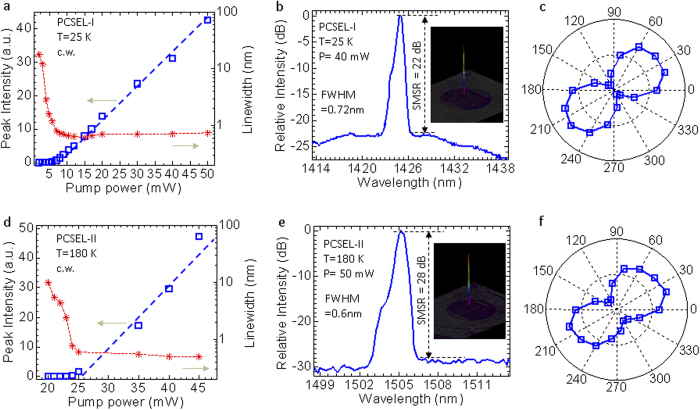
Lasing characteristics. (**a**) Lasing power and linewidth versus input pump power for PCSEL-I at T = 25 K. (**b**) Lasing spectral output (plotted in semi-log scale) above the pumping threshold (Inset: far-field image). (**c**) Measured polarization properties above threshold at λ = 1424 nm. (**d**) Lasing power and linewidth versus input pump power for PCSEL-II at T = 180 K. (**e**) Lasing spectral output (plotted in semi-log scale) above the pumping threshold (Inset: far-field image). (**f**) Measured polarization properties above threshold at λ = 1505 nm.

Devices are mounted inside a cryostat and characterized with a monochrometer-based micro-photoluminescence (μ-PL) set up (see Methods section for more details). We report here two groups of PCSEL on Si devices (PCSEL-I and -II) operating at T = 25 K and T = 180 K, respectively. The L-L plot (light output for different input light pump powers) and the corresponding spectral linewidths are shown in Fig. 2(a) for PCSEL-I operating at T = 25 K. The threshold pump power is ~6 mW, or 0.1 kW/cm^2^. The measured spectral linewidths are reduced from ~12 nm (below threshold) to 0.7 nm (above threshold). Shown in Fig. 2(b) is the measured lasing spectrum above threshold. Single mode lasing was observed at around 1424 nm with a narrow linewidth of 0.7 nm, which is essentially limited by the measurement set-up. The side mode suppression ratio (SMSR) reaches ~22 dB for the pump power P = 40 mW. Also shown in Fig. 2b inset is a measured far-field image with collimated circular single-mode output. Additionally the lasing output shows one dominant polarization, as shown in Fig. 2(c).

The measured results for PCSEL-II designed for the T = 180 K operation are presented in Fig. 2(d–f). A pumping threshold of 25 mW was observed with the linewidths reduced from ~10 nm to 0.6 nm. The measured single mode lasing spectrum and far-filed image are displayed in Fig. 2(e) where one can see a SMSR of 28 dB at a pumping power of 50 mW. Correspondingly, the output is also polarized.

**Figure 3:**
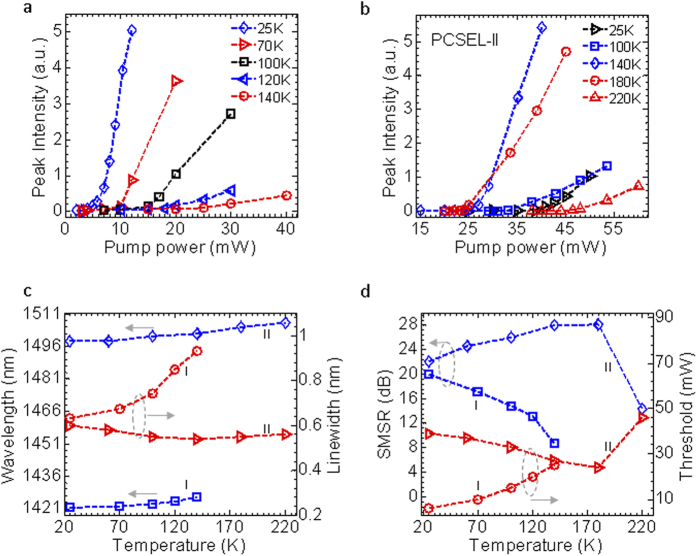
Temperature dependent properties. (**a**) Light-light (L-L) output curves for PCSEL-I. (**b**) Light-light (L-L) output curves for PCSEL-II. (**c**) The lasing peak wavelength locations and the corresponding linewidths as a function of temperature for both PCSEL-I and –II. (**d**) The side mode supersession ratios (SMSRs) and the pumping thresholds as a function of temperature for both PCSEL-I and -II.

Temperature-dependent characteristics are also investigated for both devices. Shown in Fig. 3(a,b) are the measured L-L plots at different operation temperatures for PCSEL-I and PCSEL-II devices. For PCSEL-I, the design was optimized for device operation at T = 25 K. Single mode lasing operation was obtained up to T = 140 K. However, threshold increases with the increase of the operation temperature. For PCSEL-II, on the other hand, the design was optimized for device operation around T ~ 180 K. Lasing thresholds increase with the decrease or increase of the temperature, due to the detuning of the cavity resonance and MQW emission peak. The temperature-dependent lasing characteristics are summarized in Fig. 3(c,d). For PCSEL-I, with the increase of temperature (T) from 25 K to 140 K, the lasing peak red-shifted from 1421.8 nm to 1426.55 nm, with lasing linewidth increased slightly from 0.6 nm to 0.95 nm. The lasing peak shifts at a rate of ~0.041 nm/K. The speed of cavity mode shift is much lower than the QW PL peak shift rate at 0.43 nm/K, as reported earlier^26^. It is also lower than that of the conventional VCSEL (~0.1 nm/K)^28^. The SMSR was also reduced with the increase of the temperature, with the lasing threshold increased from 6 mW to 25 mW,.

For PCSEL-II, with the change in temperature (T) from 25 K to 220 K, the lasing peak red-shifted from 1496.5 nm to 1507 nm, with lasing linewidth changing slightly around 0.6 nm. The lasing peak shifts at a rate of ~0.054 nm/K. Both SMSR and threshold degrade with the change in temperature away from the optimal operation temperature of 160–180 K. It is worth noting that an increase in the coupling between QW and Si-PC cavity by reducing the separation between the QW layer and Si-PC layer results in a reduced threshold and better lasing performance. With further optimization in the coupling design, it is feasible to achieve lasing at room temperature, with much reduced threshold and much higher output.

## Discussions

**Figure 4:**
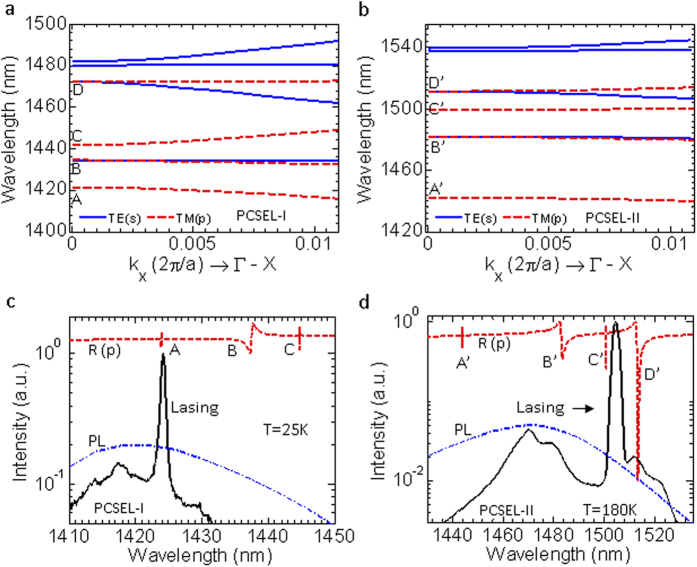
Theoretical analysis and comparison with experiment. (**a**) Photonic band diagram along Γ-X direction of Si-PC bandedge laser cavity structure for PCSLE-I. (**b**) Photonic band diagram for PCSLE-II. (**c**) Simulated reflection spectrum (red-dashed line), the measured laser emission spectrum (solid black line), and the QW PL spectrum (blue-dashed line) for PCSEL-I at T = 25 K. (**d**) Simulated reflection spectrum (red-dashed line), the measured laser emission spectrum (solid black line), and the QW PL spectrum (blue-dashed line) for PCSEL-II at T = 180 K.

To better understand the underlying physics of these two PCSEL devices, the experiment results are also correlated to theoretical and numerical analysis. First, the photonic bands are calculated from the Fano/guided resonances in the simulated reflection spectra. In Fig. 4(a,b), the eight bands are displayed in a small in-plane wave vector k_//_ range from 0 to ~0.01 (2π/a) with blue-solid lines for the transverse electric (TE, s) and red-dashed lines for the transverse magnetic (TM, p) polarizations, for PCSEL-I and -II respectively. One can see that all of these bands are very flat at the edges close to Γ point, which indicates very small group velocity and longer light-matter interaction time for these bandedge modes. Figure 4(c,d) show the simulated reflection spectra (the incident angle θ = 0.2° is used here) and measured lasing spectra for PCSEL-I and -II, respectively. Broadband PL emission spectra are also shown as references at these two different operation temperatures. These measured lasing modes correlate well with the TM guided modes (modes A and C′) in the reflection spectra for these two devices, respectively.

In order to determine why the lasing occurs at TM mode A and C′ for PCSEL-I and -II, respectively, the gain threshold (***g***_***th***_ = ***α/f***_***c***_) is investigated according to the field confinement factor (***f***_***c***_) at MQW and the loss ***α*** (or quality factor, ***Q***). Since the device is large enough with the size around 150 μm × 150 μm, which is more than 300*a* × 300*a*, it can be assumed that the edge scatting is not the dominating loss. Here only the radiation loss is considered for the infinite cavity with the relationship of ***α*** ***≈*** ***2π/aQ***^29^. The gain threshold ***g***_***th***_ values of the four band edge modes A, B, C, and D in PCSEL-I, A′, B′, C′ and D′ in PCSEL-II are estimated to be 0.92 cm^−1^, 190.4 cm^−1^, 6.96 cm^−1^, 444.9 cm^−1^, 1.61 cm^−1^, 390.4 cm^−1^, 3.79 cm^−1^, and 536.2 cm^−1^, respectively. Further information on the calculation can be found in the Supplementary Information (SI) Section. As we can see, the modes A and A′ have the lowest g_th_ in PCSEL-I and -II, respectively, which are expected to be the lasing modes. In experiments, the mode A in PCSEL-I lases as expected, while in PCSEL-II, the lasing is mode C′, instead of mode A′. The reason can be attributed to the fabrication imperfection, such as III-V/Si-PC interface defect or unflatten at QW membrane edge. Since the modes A′ and C′ have similar g_th_, we observed lasing from mode C′.

In addition, from the band structure, one can see that the mode separation is very large, due to the high refractive index contrast above and below the cavity, as compared to the thicker cavity (a few micrometers)^21^, which is highly desirable in order to achieve a single wavelength operation. This larger mode separation also indicates the stronger optical coupling in this kind of PCSEL cavities. And the coupling is further improved by putting a thicker Si-PC closer to the active region, as designed in PCSEL-II, due to higher optical confinement factor within Si-PC layer (see SI section). Comparing with PCSEL-I, the enlarged mode separation in PCSEL-II provides a stronger optical feedback, which results in a lower pump threshold, a narrower linewidth, and a higher SMSR as observed in experiments.

### Summary

In conclusion, employing membrane transfer and printing technique, hybrid Si-PC bandedge surface emitting lasers are demonstrated with optical pumping. With the careful cavity design and the control of the transfer process, single mode lasing was achieved with excellent linewidth ~6 Å and side mode suppression ratio of 28 dB. According to the numerical analysis, the experimental results agree well with the theoretical expectations. These results indicate that the combination of the transfer printing process and the photonic crystal bandedge cavity offers a simple and flexible method to realize single mode high power lasers for large-scale silicon-based photonic integration. Further optimization of the cavity design and improvement of the fabrication quality can lead to the realization of electrically pumped lasers operating under room temperature with potential 2D multi-wavelength arrays for on-chip multi-layer WDM systems with the desired spectral, spatial, and energy-efficiency.

## Methods

Device design is based on the PCSEL principle by utilizing the bandedge modes in the vicinity of the second order Γ points^30^. These modes correspond to the Fano/guided resonance modes (peaks or dips) in the reflection or transmission spectra of the cold cavity. The cavity structure parameters are optimized by tuning these Fano resonance modes to match with the MQW emission peak. All of the simulations used the Fourier Modal Method with Stanford Stratified Structure Solver (S^4^) software package^31^. In the simulation, a periodic boundary condition is used, corresponding to the cavity with in-plane infinite size.

Device characterization are carried out by mounting the devices inside a cryostat and characterized with a monochrometer-based micro-photoluminescence (μ-PL) set up. See the details in the third section of Supplementary Information (SI).

## Additional Information

**How to cite this article**: Zhao, D. *et al.* Printed Large-Area Single-Mode Photonic Crystal Bandedge Surface-Emitting Lasers on Silicon. *Sci. Rep.* **6**, 18860; doi: 10.1038/srep18860 (2016).

## Supplementary Material

Supplementary Information
