# Supplementary material for: Printed Large-Area Single-Mode Photonic Crystal Bandedge Surface-Emitting Lasers on Silicon
Source: Sci Rep. 2016 Jan 4;6:18860. doi: 10.1038/srep18860 (PMC4698743; doi:10.1038/srep18860)
Supplement: Supplementary Information [file srep18860-s1.pdf]

Printed Large-Area Single-Mode Photonic Crystal Bandedge Surface-Emitting Lasers on Silicon

Deyin Zhao[1a], Shihchia Liu[1a], Hongjun Yang[1], Zhenqiang Ma[2], Carl Reuterskiöld-Hedlund[3], Mattias Hammar[3], and Weidong Zhou[1*]

[1]*Department of Electrical Engineering, University of Texas at Arlington, TX 76019, USA*

[2]*Department of Electrical and Computer Engineering, University of Wisconsin-Madison, WI 53706, USA*

[3]*KTH-Royal Institute of Technology, School of Information and Communication Technology, Electrum 229, 164 40 Kista, Sweden*

[a]*These authors contributed equally this work*

**Corresponding author: email: wzhou@uta.edu*

Supplemental Information (SI)

Supplementary Information (SI)

I. Lasing principle and cavity design of PCSEL

The principle of the lasing modes in the photonic crystal surface emitting laser (PCSEL) cavities is based on the band edge modes of two-dimensional (2D) PC close to the second order Γ points[1-3], where the group velocity of these modes is close to zero. In such 2D PC cavities, the periodic patterned structure is designed close to the unpatented active region to provide strong in-plane distribution feedback (DFB) and the standing wave is formed in multidirectional directions due to 2D DFB effect[1]. Simultaneously, the in-plane propagating waves are diffracted toward the vertical direction (i.e., the surface emission) by the PC structure itself due to the first-order Bragg diffraction. It is because of this multidirectional Bragg diffraction in these 2D PC structures, the cavity mode can lase over a large 2D area and gives a high power output[4,5].

According to the second Bragg condition ($\boldsymbol{a}=\lambda/n_{eff}$), the lattice constant $\boldsymbol{a}$ of the PCSEL cavity is chosen to be around 480 nm to realize the surface emitting output for the emission wavelength close to the communication wavelength range of 1550 nm. The approximate refractive index n_{eff} of the whole cavity (InGaAsP MQW active layer and Si-PC) is around 3.2 according to the average dielectric constant in the initial design. On the other hand, in order to reduce the vertical radiation loss to obtain high Q and low threshold, the air hole radius is relatively small: $\boldsymbol{r} = 0.2\boldsymbol{a}$ for PCSEL-I and $0.15\boldsymbol{a}$ for PCSEL-II. To match the cavity mode with the MQW emission peak, the PCSEL cavity structure parameters are optimized by tuning the Fano/guided resonance modes of the cold cavities, which can correspond to the peaks or dips in the reflection or transmission spectra. All the spectral simulations are employed by using the Fourier Modal Method with Stanford Stratified Structure Solver (S^4) software package [6], In the simulation, a periodic boundary condition is used since the cavity in-plane size is over 100 μm or 200 periods, which can be regarded as infinity.

II. Theoretical and numerical analysis of PCSEL

The schematic of the modeled real PCSEL devices is shown in Fig. S1(a), where the whole cavity sits on an Si substrate with an oxide layer of thickness 400 nm in between. The total thicknesses of the active layer (t_1) and Si-PC (t_2) are 545nm and 527nm for PCSEL-I and –II, respectively. Different from the cavity structure where PC is embedded inside the thicker cavity[1,4], pure 2D approximation in band calculation is not entirely accurate for the cavity with high refractive index contrast to the surrounding material. Here, the photonic bands of the real devices are calculated from the Fano/guided resonances peaks or dips in the simulated reflection or transmission spectra according to the relation between in-plane wave vector $\boldsymbol{k}_{//}$ and the incident angle θ, $k_{//} = 2\pi / \lambda \cdot \sin\theta$ [7], where λ is the wavelength in free space. By changing the incident angle from the surface normal direction (z-axis) to in-plane along the x-axis direction, the photonic bands in Γ - X direction can be obtained, with Γ point corresponding to the normal incidence angle θ = 0°. Here we mainly focus on a small value range of incident angle θ from 0° to 2°, which corresponds to the $k_{//}$ range from 0 to ~0.01 (2π/a) in the vicinity of the band edge. For θ = 0°, the non-degenerate modes at Γ point are obtained from spectra of the incident angle θ = 0.01° because the non-degenerate modes cannot be directly excited by the outside normal incident light due to the symmetric property [8]. Owing to the same reason, the simulated reflection spectra with θ=0.2° is shown in Figure 4(c) and (d) in the main context. The calculated band structures with normalized frequency are plotted in Fig. S1(b) and (c) for PCSEL-I and -II, respectively. One can clearly see the flat bands close to Γ point and the larger mode separation for the non-degenerate TM modes, ~0.005 in PCSEL-I and 0.013 in PCSEL-II. By comparing with the mode separation in the thicker cavity, the larger mode separation in the thin film cavity attributes to the high contrast refractive index to the surrounding. On the other hand, the index contrast is further increased by using thick Si-PC and thinner active layer in PCSEL-II so that the mode separation is

enlarged further by comparing with PCSEL-I. Therefore, single mode lasing are achieved in this kind of PCSEL cavities.

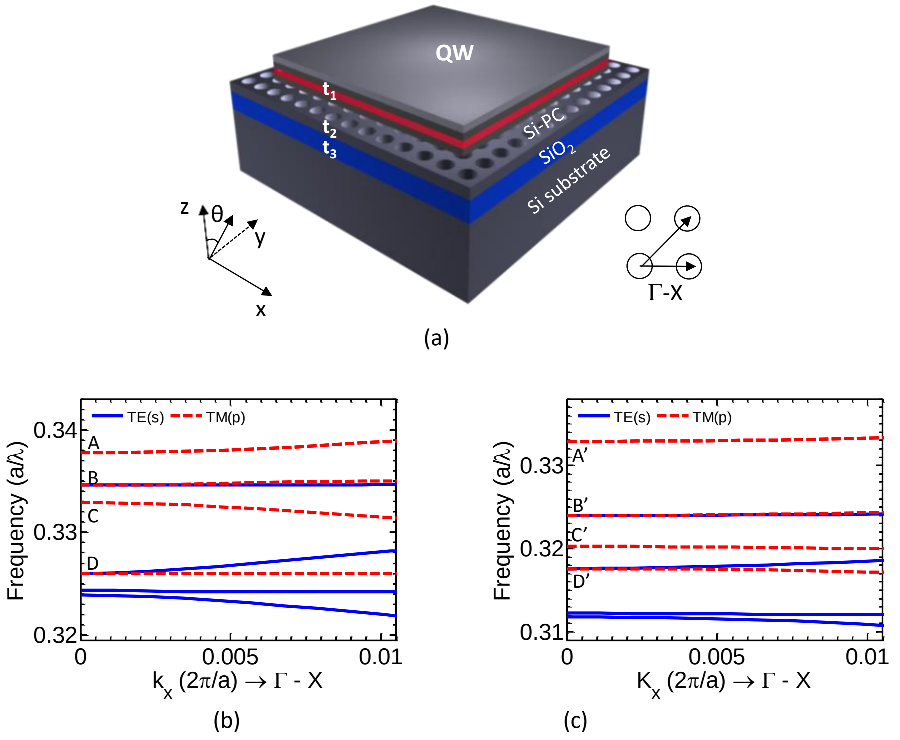

Figure S1. Simulated photonic crystal dispersion properties. a, 3D sketch of the modeled structure of hybrid III-V/Si PCSEL cavity on Si substrate. **b,** Photonic band diagram along Γ-X direction of Si-PC bandedge laser cavity structure for PCSLE-I with normalized frequency. **c,** Photonic band diagram for PCSLE-II.

The field profiles of mode A and mode C' are displayed in Fig. S2 (a) and (b), respectively, where the integrated H-field intensity of the whole cavity along the z-axis direction is plotted together with the cavity index profile. The cross-section views of H-field intensity of modes A and C' are shown in Fig. S2 (c) and (d), respectively. One can see the field is strongly confined inside the thin cavity due to high index contrast below and above the laser cavity. The confinement factor for the TM band modes is calculated according to the

magnetic field distribution $f_c = \int_{QW} H^2 dv / \int_{cav} H^2 dv$ [9]. The f_c values of the modes A (A'), B (B') , C (C') and D (D') are calculated for these two devices PCSEL-I (II). Their quality factors (Qs) are obtained by Fano fitting[10] the guided resonances in reflection spectra. Here only the radiation loss is considered for the infinite cavity with the relationship $\alpha \approx 2\pi/aQ$ [11]. Therefore the gain threshold is equal to $g_{th}=\alpha/f_c$. Including f_c and Q, the consequent values of the quantities of α and g_{th} are summarized in Table S1. From the table, we know the final laser oscillation is expected to occur at mode A in PCSEL-I and mode A' in PCSEL-II with the lowest gain threshold, - In experiments, we observed the mode A in PCSEL-I lases, while in PCSEL-II, the lasing is mode C', instead of mode A' due to the reasons including the fabrication imperfect and similar g_{th} between modes A' and C'.

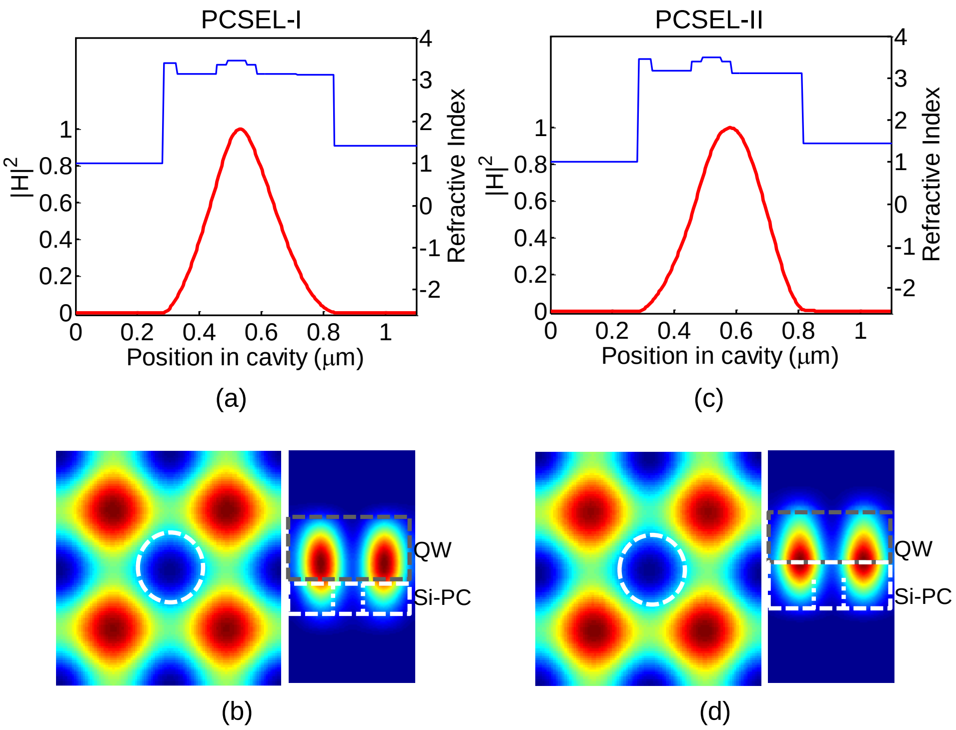

Figure S2. Cavity design and simulated field distributions. The simulated H-field intensity distributions of the hybrid III-V/Si PCSEL cavity modes: In-plane integrated H-field intensity distribution along z-axis direction (cavity surface normal), **a,** Mode A in PCSEL-I and **b,** Mode B' in PCSEL-II; Cross section views of field distributions in the xy plane (right) and xz

plane (left), **c,** Mode A in PCSEL-I and **d,** Mode C' in PCSEL-II. The circular and rectangular dashed lines in the field images indicate the air hole in Si-PC and the boundary of the QW and Si-PC in the cavity.

Table S1. The calculated values of the quantities $\boldsymbol{f_c}$, $\boldsymbol{Q}$, $\boldsymbol{\alpha}$ and $\boldsymbol{g_{th}}$.

Mode	f_c (%)	Q	α (cm^{-1})	g_{th} (cm^{-1})
A	24.4	5.86e5	0.22	0.92
B	19.7	3.49e3	37.5	190.4
C	21.7	8.67e4	1.51	6.96
D	17.3	1.70e3	76.96	444.9
A'	23.64	3.45e5	0.38	1.61
B'	19.85	1.69e3	77.5	390.4
C'	20.6	1.67e5	0.78	3.79
D'	18.5	1.32e3	99.2	536.2

In addition, comparing the field distributions of PCSEL-I and PCSEL-II in Fig. S2, one can see more field distributed in the Si-PC region in PCSEL-II than that in PCSEL-I. The confinement factors of Si-PC ($\boldsymbol{f_{c,PC}}$) are calculated to be around 4.56% and 46% for PCSEL-I and PCSEL-II, respectively. The much improved field confinement in Si-PC region indicates much stronger optical feedback in PCSEL-II. Consequently, better lasing performances are achieved in PCSEL-II than PCSEL-I in experiments, including the lower threshold, narrower linewidth, and higher SMSR, which agrees well with theoretical design and analysis.

III. Device characterization setup

Device characterization are carried out by mounting the devices inside a cryostat and characterized with a monochrometer-based micro-photoluminescence (μ-PL) set up, as shown in Fig. S3. A continuous wave (c.w.) green (532 nm) laser is used as the pump source incident from the surface normal direction via a long working distance objective lens (M=10 x). The diameter of the excitation spot is around 110 μm. The emitted light was also collected from the same objective lens and separated with a cold mirror. The collected emission light was

sent into the monochrometer and detected with a thermoelectric-cooled InGaAs photodetector. A chopper and lock-in amplifier are used for the spectral acquisition of lasing output. An infrared (IR) camera is mounted at the other output port of the monochrometer to acquire the far field image. A polarizer is placed in front of the entrance of the monochrometer to investigate the polarization properties of the emission beams.

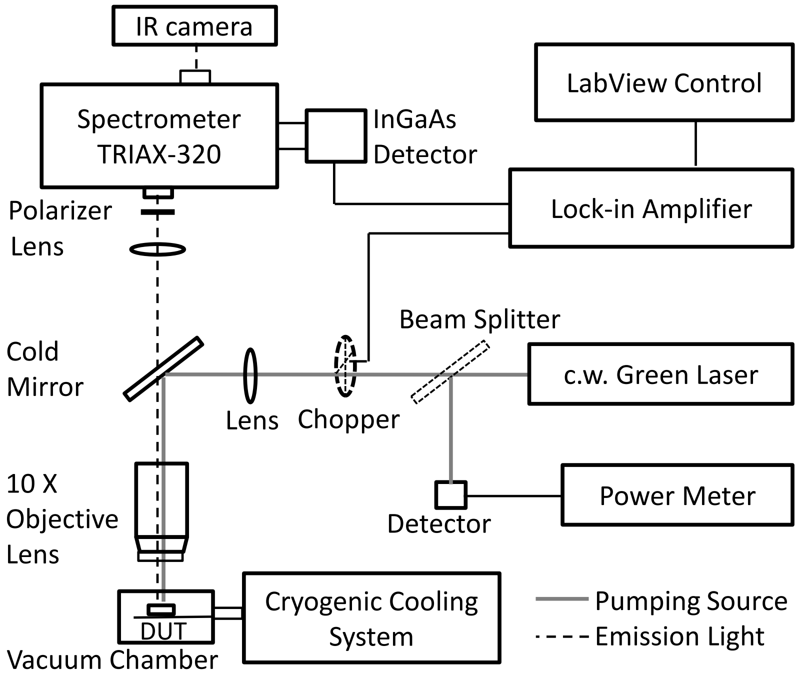

Figure S3. The schematic illustration of the Micro-PL system.

References

1 Imada, M., Chutinan, A., Noda, S. & Mochizuki, M. Multidirectionally distributed feedback photonic crystal lasers. *Phys Rev B* **65**, 195306 (2002).

2 Sakai, K. *et al.* Lasing band-edge identification for a surface-emitting photonic crystal laser. *Ieee J Sel Area Comm* **23**, 1335-1340, doi:Doi 10.1109/Jsac.2005.0851205 (2005).

3 Meier, M. *et al.* Laser action from two-dimensional distributed feedback in photonic crystals. *Appl Phys Lett* **74**, 7-9, doi:Doi 10.1063/1.123116 (1999).

4 Hirose, K. *et al.* Watt-class high-power, high-beam-quality photonic-crystal lasers. *Nature Photonics* **8**, 406-411, doi:DOI 10.1038/nphoton.2014.80 (2014).

5 Chua, S. L., Lu, L., Bravo-Abad, J., Joannopoulos, J. D. & Soljacic, M. Larger-area single-mode photonic crystal surface-emitting lasers enabled by an accidental Dirac point. *Optics letters* **39**, 2072-2075, doi:Doi 10.1364/Ol.39.002072 (2014).

6 Liu, V. & Fan, S. S4: A free electromagnetic solver for layered periodic structures. *Computer Physics Communications* **183**, 2233-2244 (2012).

7 Lee, J. *et al.* Observation and differentiation of unique high-Q optical resonances near zero wave vector in macroscopic photonic crystal slabs. *Phys Rev Lett* **109**, 067401 (2012).

8 Fan, S. & Joannopoulos, J. D. Analysis of guided resonances in photonic crystal slabs. *Phys Rev B* **65**, 235112 (2002).

9 Adams, M. An introduction to optical waveguides. *Chichester-New York* (1981).

10 Miroshnichenko, A. E., Flach, S. & Kivshar, Y. S. Fano resonances in nanoscale structures. *Reviews of Modern Physics* **82**, 2257 (2010).

11 Rosenblatt, D., Sharon, A. & Friesem, A. A. Resonant grating waveguide structures. *Ieee J Quantum Elect* **33**, 2038-2059, doi:Doi 10.1109/3.641320 (1997).
